# Zooplankton network conditioned by turbidity gradient in small anthropogenic reservoirs

**DOI:** 10.1038/s41598-022-08045-y

**Published:** 2022-03-10

**Authors:** Anna Maria Goździejewska, Marek Kruk

**Affiliations:** 1 Faculty of Geoengineering, University of Warmia and Mazury, Oczapowskiego 5, 10-719 Olsztyn, Poland; 2 Faculty of Mathematics and Computer Science, University of Warmia and Mazury in Olsztyn, Słoneczna 54, 10-710 Olsztyn, Poland

**Keywords:** Ecology, Environmental sciences, Limnology

## Abstract

Water turbidity can significantly influence interspecific interactions in aquatic ecosystems. We tested the hypothesis that the turbidity gradient significantly differentiates the dynamics, significance and type of relationships in the structure of zooplankton communities colonizing mine pit reservoirs. The interactions between zooplankton species were evaluated by network graph analysis for three water turbidity classes: high turbidity (HT), moderate turbidity (MT) and low turbidity (LT). The HT network was most cohesive, and it was controlled by taxa grazing on various food sources within one ecological niche (*Polyarthra longiremis*, *Brachionus angularis*, *Cyclops vicinus*, *Codonella cratera*) and the positive and negative relationships between them were balanced. The MT biocenotic network was composed of three sub-networks connected by nodes with high communication attributes (*Polyarthra vulgaris*, *Bosmina longirostris*, *C. vicinus*), and antagonistic interactions (predation and competition) were less important. The LT network was most heterogeneous, and *Daphnia cuculllata* exerted the strongest influence on the network’s structure by forming numerous positive (coexistence with predators) and negative (interference competition with microphagous rotifers) interspecific relationships. The study provides new information about the ecology of aquatic ecosystems, that are disturbed by changes in water turbidity.

## Introduction

Zooplankton play a fundamental role in the structure and functioning of species interaction networks in aquatic ecosystems communicating the primary producers-level (phytoplankton) and higher-order consumers (ichthyofauna)^1–3^. Zooplankton are often regarded as bioindicators of water quality due to their widespread abundance, small size, high reproductive rate, and early and sensitive responses to changes in environmental parameters (pollution, eutrophication, changes in water level, turbidity)^4–8^. Planktonic animals deploy various ecological strategies, they are characterized by phylogenetic distinctness and can be dispersed passively across large areas, which is why they are often used to test ecological theories and develop ecosystem (prognostic) models^9–14^. The structure of plankton communities is determined by abiotic environmental conditions (filters), and it is characterized by a specific species composition and functional traits^15–18^. This provides a basis for forming interspecific relationships, which in the case of zooplankton consist mainly of competitive exclusion and predation^19,20^. The structure of the interactions between the abiotic environment and the features of zooplankton assemblages is frequently analysed to obtain reliable information about the current status of aquatic ecosystems and to forecast future changes.

Water turbidity is a factor that significantly differentiates interspecific relationships in aquatic ecosystems^21,22^. Turbidity is an optical property that determines the degree of light scattering and absorption, thus it indicates the difficulty for the light rays to penetrate deep into the water. Water turbidity is caused by the presence of suspended particles (clay and sand), fine particles of organic and inorganic matter, soluble colloidal and humic substances, planktonic organisms and bacteria^23^. Therefore, turbidity can be an indicator of various biological, physical and chemical processes, depending on the origin, concentration and type of suspended particles^24–28^. Turbidity is also an important parameter of water quality and ecosystem productivity. Higher turbidity is often associated with an increase in water color and temperature, phytoplankton primary production, algal blooms and bacterial decay^29,30^. High turbidity is indicative of low water transparency and a limited euphotic (productive) zone, and it can significantly affect the feeding efficiency, development and abundance of filter-feeding zooplankton^31–34^, as well as limit the foraging success of fish that locate prey visually^21,22,35,36^. High turbidity accompanied by a low concentration of suspended solids could be indicative of small particle size and a high share of nanoparticles^37^. Due to a high active surface area to volume ratio, nanoparticles are highly reactive, and their chemical composition could pose a risk of toxicity for aquatic organisms, including zooplankton^38–41^. Therefore, the turbidity gradient differentiates the species composition of zooplankton communities, and it can also affect the type and strength of interspecific interactions, including predation and competition^42,43^.

Most in situ studies investigating the effects of high water turbidity on zooplankton have focused on periodic disturbances caused by intense weather phenomena such as gale-force winds, torrential rain^4,5,8^ or hydrodynamic processes such as tides^44^. Few studies have analysed the correlations between zooplankton communities and high water turbidity associated with human activity^29,30,37^. Their results indicate that an increase in turbidity was accompanied by changes in the taxonomic structure, abundance, biomass and functional traits of zooplankton (size, feeding strategy). However, the response of zooplankton communities to water turbidity has never been analysed from a structural perspective, i.e. by investigating interspecific relationships, including the extent to which individual taxa influence the cohesion and other properties of planktonic networks. The resulting knowledge is particularly important in reservoirs that are subjected to constant anthropogenic pressure, including natural water bodies as well as artificial reservoirs that are built and utilized for specific purposes (economic, social, recreational). In this context, a knowledge of zooplankton network functions can have practical implications, for example for managing fisheries or reclaiming water bodies through biomanipulation. Understanding the zooplankton networks can be used for waters systems monitoring due to climate change. The predicted increases in wind speed and wave high in the global scale^45^ may cause an increase of destabilization and turbidity of the water column due to resuspension of sediments in shallow lakes, and changes in their biological structure^8^.

The artificial reservoirs located in the vicinity of Bełchatów and Szczerców coal strip mines in Central Poland are a good example of the above. These reservoirs were created by draining strip pits. They are used mainly as settlement ponds, but they are also popular destinations for recreational fishing. The limnological and hydrological characteristics of reservoirs, that are regularly inspected and steadily supplied with suspended matter create unique opportunities for analyzing the structure of planktonic communities in situ^37,46,47^. Previous studies conducted in 2012–2013 revealed that the size and chemical properties of suspended solids significantly affect the taxonomic structure and functions of zooplankton communities^37,47^. The present study is a continuation of the previous research into the influence of suspended solids (expressed in turbidity units) on zooplankton dynamics, presented in the form of a network of interspecific relationships with the use of network graph analysis. The network graph model is a method that supports the identification and assessment of interspecific relationships such as mutualistic interactions (positive correlations), coexistence within guilds, and limiting factors (negative correlations) such as predation and competition^12,48,49^.

Zooplankton networks were analysed based on the biomass parameters of crustaceans, rotifers and protozoans examined between 2014 and 2016. We assumed that positive interactions between two taxa would be correlated with an increase in their biomass as the effect of consumer guilds that independently exploit the same resources^50^. In turn, negative interactions between species (biomass parameters) would be indicative of grazing on phytoplankton, predation or interference competition.

The aim of the study was to determine how the interspecies relationships of zooplankton change due the water turbidity gradient, and how turbidity affects the stability and functionality of the zooplankton network. We hypothesized that the turbidity gradient significantly differentiates the dynamics of zooplankton species and strongly modifies their competitive equilibrium, thus influencing the role played by individual taxa and interspecific relationships in network cohesion. To validate the research hypothesis, eight reservoirs were divided into three groups that differed significantly in water turbidity. We assumed that the significance and strength of interspecific relationships (with an equal number of positive and antagonistic biocenotic interactions) and, consequently, the cohesion and centrality of the network would increase with a rise in turbidity. In turn, a decrease in turbidity would be correlated with decentralization and fragmentation of the network, and with weakening of interspecific interactions in zooplankton communities.

## Results

### Environmental variables and zooplankton distribution along the turbidity gradient

Significant differences in the physical and chemical parameters of water were noted between turbidity classes HT, MT and LT. In addition to significant variation in mean turbidity values (31.8, 18.7 and 12.6 NTU respectively), turbidity classes also differed significantly in water color, total suspended solids, and chlorophyll *a* levels (Table 1). Turbidity was positively correlated with color (*r* = 0.521) and total suspended solids (Tot susp *r* = 0.510), and it was negatively correlated with STD (*r* = − 0.553). The remaining variables (Temperature, DO, pH, TP, TN) did not differ significantly across the evaluated turbidity classes.

**Table 1:** Water quality and zooplankton parameters across the studied turbidity classes (mean ± SD).

	LT		MT		HT		*P*
	$\overline{x}$	± SD	$\overline{x}$	± SD	$\overline{x}$	± SD	
**Physical and chemical parameters of water**							
Turbidity (NTU)	12.58^a^	7.34	18.67^b^	9.99	31.83^c^	17.87	0.000
SDT (m)	1.18^a^	0.38	0.74^b^	0.20	0.54^c^	0.14	0.000
Color (Hazen)	10.10^a^	6.99	18.03^b^	13.51	23.87^b^	19.10	0.000
Tot susp (mg/l)	4.35^a^	3.05	6.84^ab^	5.60	9.03^b^	6.99	0.000
In susp (mg/l)	1.27^a^	1.26	2.76^ab^	3.02	3.24^b^	3.49	0.003
Org susp (mg/l)	3.08^a^	2.47	4.08^ab^	4.90	5.79^b^	5.93	0.019
Chl *a* (µg/l)	3.46^a^	4.43	4.49^ab^	2.97	6.16^b^	6.81	0.027
Temperature (°C)	17.44	3.06	16.71	4.87	17.08	4.36	> 0.05
pH	7.74	0.26	7.78	0.32	7.80	0.27	> 0.05
DO (mg/l)	8.29	1.41	8.74	1.24	8.66	0.93	> 0.05
TP (mg/l)	0.12	0.06	0.11	0.11	0.11	0.06	> 0.05
TN (mg/l)	0.22	0.10	0.28	0.12	0.30	0.12	> 0.05
**Zooplankton measures**							
Biomass (mg/l)	0.122^a^	0.269	8.02^b^	12.42	3.66^c^	6.76	0.000
Abundance (ind./l)	105.9^a^	407.7	1265.5^b^	1555.4	751.4^c^	1345.20	0.000
Number of species (ind.)	17^a^	5	17^a^	4	15^b^	5	0.009
Shannon’s biodiversity index *H*′	1.99^a^	0.58	1.64^b^	0.39	1.62^b^	0.45	0.000
Pielou’s eveness index, *J*′	0.714^a^	0.192	0.583^b^	0.127	0.622^b^	0.175	0.001

Differences in the analysed parameters were determined by ANOVA (df = 157; *P* ≤ 0.05); values with the different superscripts are different among reservoirs by post-hoc Tukey test.

*SDT* Secchi depth, *Tot susp* total suspended solids, *In susp* inorganic suspended solids, *Org susp* organic suspended solids, *Chl a* chlorophyll *a*, *DO* dissolved oxygen, *TP* total phosphorus, *TN* total nitrogen.

The turbidity gradient significantly influenced the species richness of zooplankton. Species diversity was highest in the LT class (*H*′ = 1.99; *J*′ = 0.714), and it was significantly lower in MT and HT classes (*H*′ = 1.64 and 1.62; *J*′ = 0.583 and 0.622, respectively; Table 1). The zooplankton community was composed of 102 taxa in the LT class, and 85 taxa each in MT and HT classes. Rotifera species were dominant in all turbidity classes, and they accounted for 72–75% of all taxa. The taxonomic structure of Crustacea was more diverse, where Cladocera were predominant in the LT class (15%; 10–11% in the remaining classes), and Copepoda were predominant in the MT class (17%; 13% in the HT class; 7% in the LT class). Fifty-six taxa and forms (43%) were identified in all turbidity classes, with a predominance of juvenile copepodite stages (69% in the LT class; 98% in the MT class) and *Polyarthra longiremis* (41% in the LT class; 90% in the MT class) (Table S1).

The turbidity gradient significantly differentiated (ANOVA, Kruskal–Wallis test, *P* ≤ 0.05) the biomass distribution of 35 (26.9%) zooplankton taxa, including 24 Rotifera, 3 Cladocera, 5 Copepoda, 3 Protozoa (Table S1). As a result, total zooplankton biomass differed significantly across turbidity classes and was determined at 0.122 mg/l in the LT class, 8.02 mg/l in the MT class, and 3.66 mg/l in the HT class (Table 1).

### Network structure

**Figure 1:**
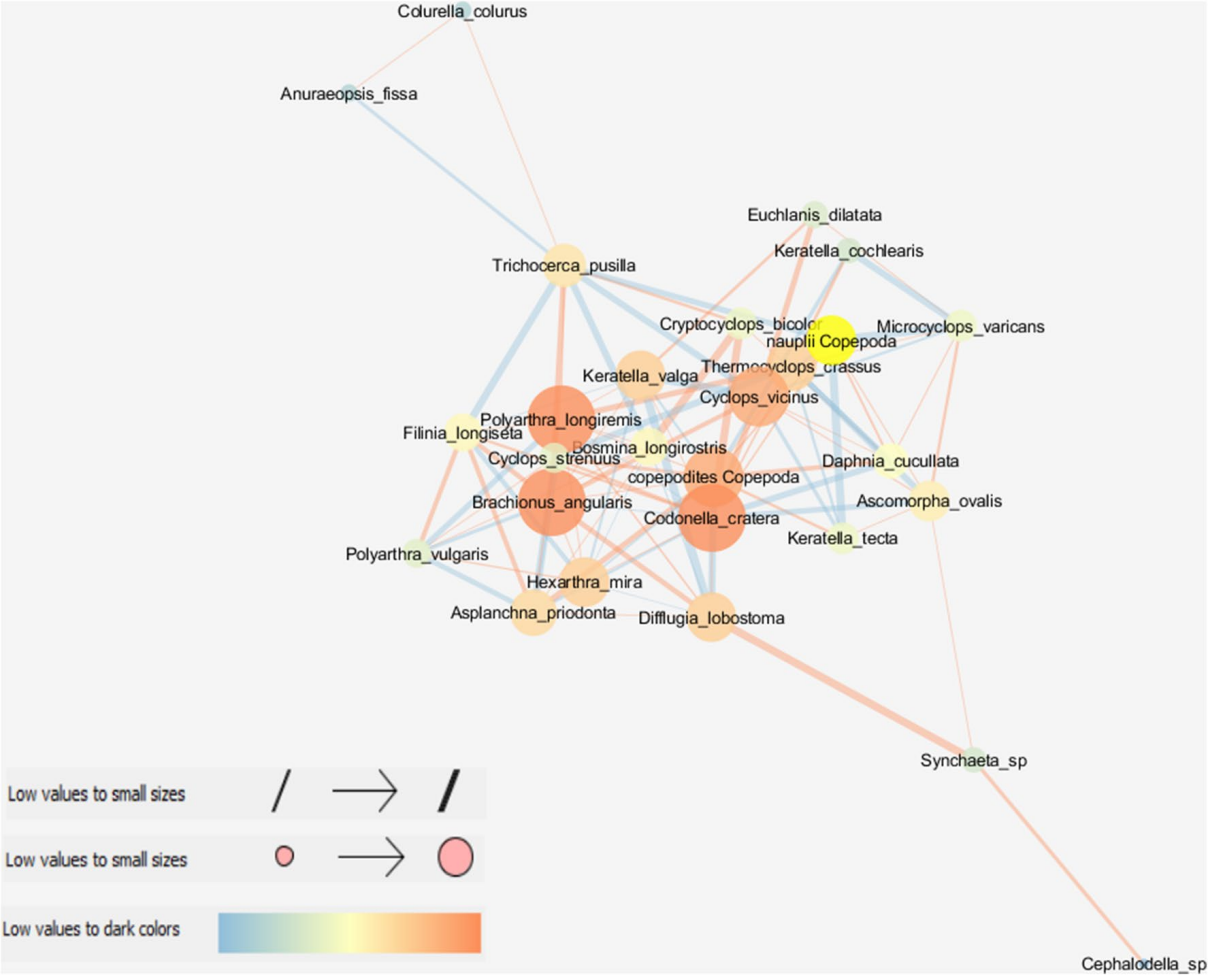
Network graph analysis of the interactions between zooplankton species in the HT class with node closeness centrality (NCC), node betweenness centrality (NBC) and edge betweenness centrality (EBC). Node size is proportional to the NCC measure; node color ranging from blue (dark) to orange (bright) is proportional to the NBC measure; edge thickness is proportional to the EBC measure. Sign of the relationship: a bright orange edge denotes positive relations between nodes, while a dark blue edge represents negative relations.

**Figure 2:**
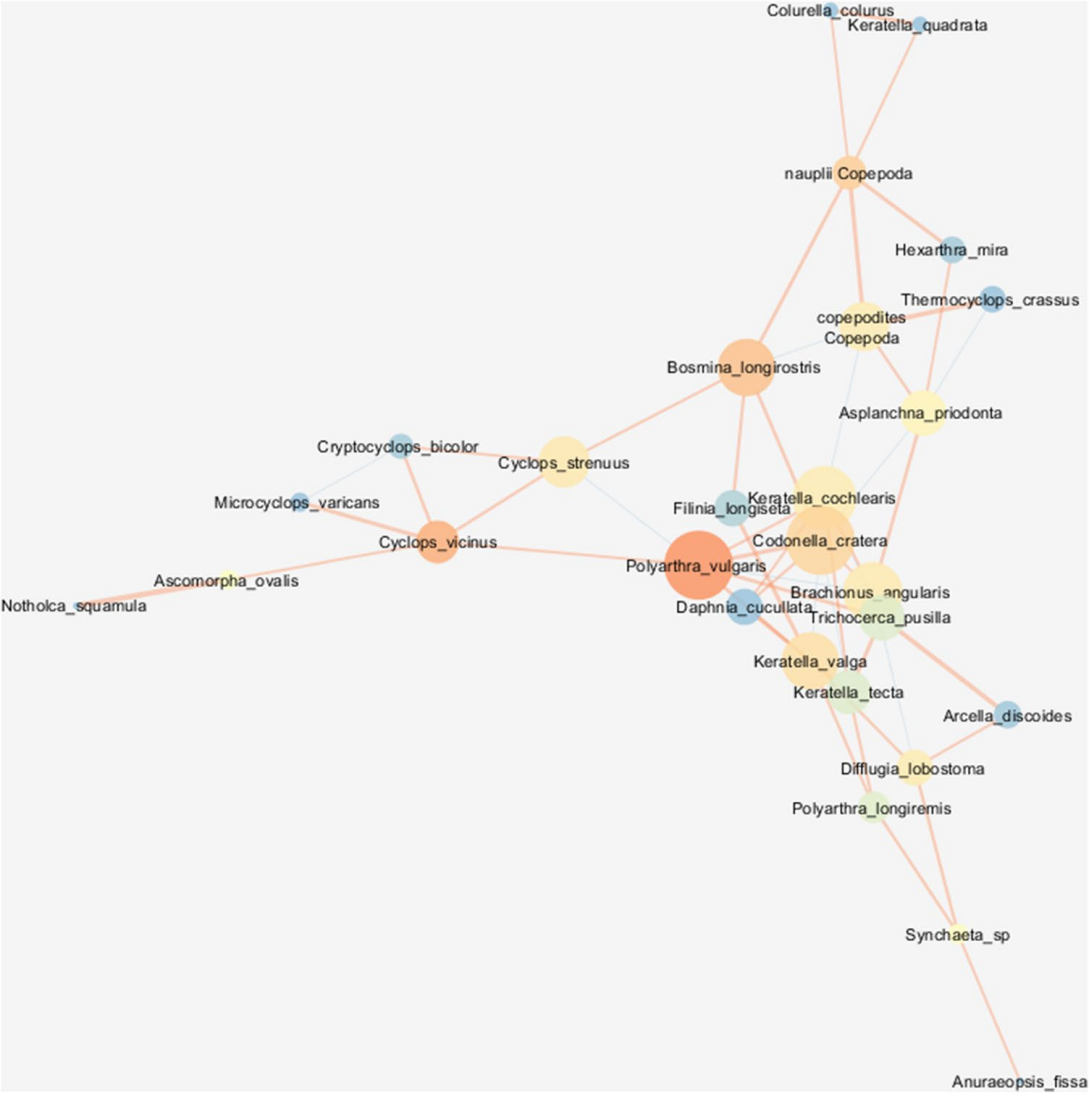
Network graph analysis of the interactions between zooplankton species in the MT class with node closeness centrality (NCC), node betweenness centrality (NBC) and edge betweenness centrality (EBC). Refer to the legend and explanations in Fig. 1 (HT class).

**Figure 3:**
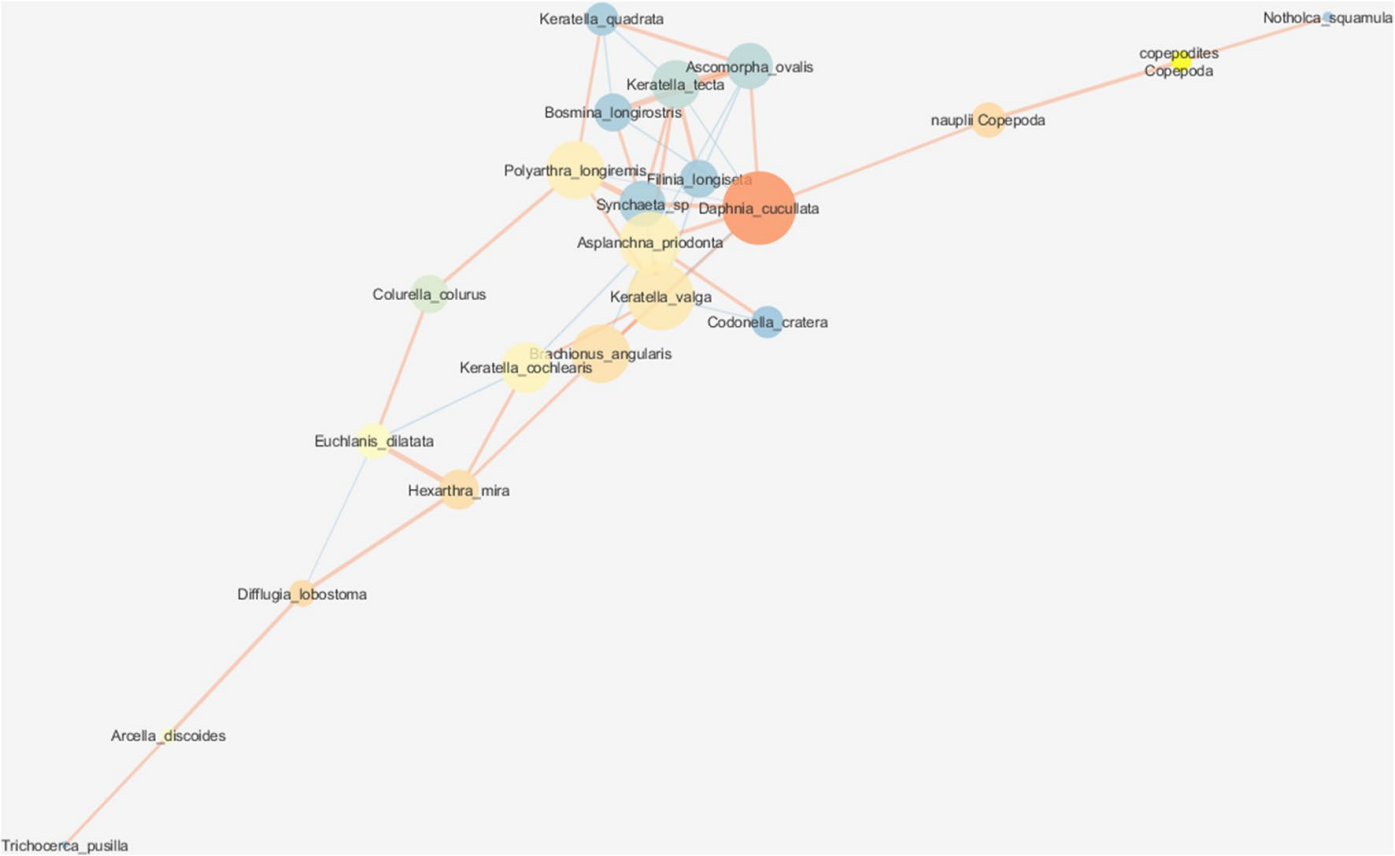
Network graph analysis the interactions between zooplankton species in the LT class with node closeness centrality (NCC), node betweenness centrality (NBC) and edge betweenness centrality (EBC). Refer to the legend and explanations in Fig. 1 (HT class).

The compared turbidity classes differed in the key metrics describing the structure of the zooplankton species interaction network. The HT network was characterized by the highest cohesion and density expressed by clustering (0.523), density (0.279) and centrality (NCC = 0.280) metrics, as well as the highest average number of neighbors (7.26) per species (node), i.e. the number of interspecific interactions (Table 2; Fig. 1). The MT network was characterized by the lowest centrality (NCC = 0.134) and density (0.135) metrics, as well as the highest values of the parameters describing communication paths between taxa, i.e. the shortest paths (756) and characteristic path length (2.95), which denote the presence of taxa that communicate with the highest number of species (Table 2; Fig. 2). The LT network was most fragmented, as indicated by the highest value of the network heterogeneity parameter (0.528) and the lowest number of the shortest paths (462) (Table 2; Fig. 3).

**Table 2:** General attributes of the zooplankton network in compared turbidity classes.

Attribute	Turbidity class		
	LT	MT	HT
Clustering coefficient	0.332	0.3	0.523
Network centralization	0.252	0.134	0.28
Shortest paths (100%)	462	756	702
Characteristic path length	2.81	2.947	2.023
Range, and average number of neighbours	2.81	3.643	7.259
Network density	0.199	0.135	0.279
Network heterogeneity	0.528	0.465	0.474

### Interspecific relationships in zooplankton networks

Node degree centrality (NDC) describes the number of direct links with a given taxon (node), and it is an important measure of interspecific relationships. The highest NDC values (more than 10 links per taxon) were noted in the HT class for *Hexarthra mira*, *Polyarthra longiremis*, *Keratella valga*, *Brachionus angularis, Filinia longiseta, Cyclops strenuus* and *Difflugia lobostoma*. The maximum NDC values were noted in LT and MT classes, 8 (*Codonella cratera*) and 6 (*Daphnia cucullata*), respectively. In the LT class, the strongest positive relationships were observed between Rotifera taxa which were characterized by the highest correlation coefficients, for example: *Asplanchna priodonta*–*K. valga* (0.867) and *Polyarthra longiremis*–*Synchaeta* spp. (0.853). Significant positive correlations were also associated with copepod species in the MT class, and cladoceran species in the HT class. The significance of competitive and predatory behaviors (negative correlations) increased in extreme turbidity classes, for example: *Bosmina longirostris*–*Keratella tecta* (− 0.605) and *P. longiremis*–*B. angularis* (− 0.450) (Figs. 1, 2, 3; Table S2).

Species characterized by the highest NCC values, i.e. taxa that exerted the greatest influence on other species, were most prevalent in the HT class, and NCC values were also highest in the HT class in comparison with the remaining turbidity classes (Table 3). In the HT class, rotifers *P. longiremis* and *B. angularis*, protozoan *Codonella cratera,* copepod *Cyclops vicinus* and juvenile copepodite stages were characterized by the highest centrality attribute values (> 0.605) (Fig. 1). The highest centrality attribute values in the LT network (NCC > 0.467) were noted in *D. cucullata*, *K. valga*, and *A. priodonta* (Fig. 3). In the MT class, the highest NCC values (> 0.42) were observed in rotifers *Polyarthra vulgaris, B. angularis* and *K. valga,* cladoceran *Bosmina longirostris,* and protozoan *C. cratera* (Fig. 2, Table 3).

**Table 3:** Zooplankton species with the highest net attribute.

	LT			MT			HT		
	NCC	NBC	NDC	NCC	NBC	NDC	NCC	NBC	NDC
**Rotifera**									
*Polyarthra longiremis*	0.456			0.342			0.634		10
*Brachionus angularis*	0.456	0.162	7	0.435	0.112	6	0.634		10
*Polyatrhra vulgaris*				0.465	0.262	5	0.456		
*Keratella valga*	0.488	0.144		0.428	0.129		0.565		10
*Asplanchna priodonta*	0.467		6	0.391			0.553		9
*Trichocerca pusilla*						5		0.169	
*Hexarthra mira*		0.172							10
*Keratella cochlearis*		0.114	6		0.107				
*Filinia longiseta*			7						10
**Crustacea**									
*Cyclops vicinus*				0.38	0.216		0.605		8
*Cyclops strenuus*					0.115	5			10
*Bosmina longirostris*	0.382		6	0.428	0.184	5	0.52		9
*Daphnia cucullata*	0.512	0.341	6	0.36		6	0.509		9
Copepodites	0.28			0.403	0.107		0.605		8
Nauplii		0.181	6		0.16				8
**Protozoa**									
*Codonella cratera*	0.35		8	0.465	0.145	5	0.634	0.114	
*Difflugia lobostoma*		0.181			0.109				10

*NCC* node closeness centrality, *NBC* node betweenness centrality, *NDC* node degree centrality.

The importance of taxa in the cohesion of the entire network, measured by node betweenness centrality (NBC) metrics was greater in moderate and low turbidity classes, because this attribute favors taxa that connect with subnets (clusters). Thus, when a network is less coherent and more fragmented, the taxa (nodes) that communicate with other network clusters play a more important role than those within the network. In the MT class, the highest NBC values (> 0.200) were noted in raptorial taxa *P. vulgaris* and *C. vicinus*, but *B. longirostris*, copepod nauplii and *C. cratera* were also characterized by high NBC values (> 0.140). The most heterogeneous LT class network favored mostly *D. cucullata* (0.341), but *Difflugia lobostoma*, *H. mira* and copepod nauplii were also highly interactive species (NBC > 0.160). In turn, in the most cohesive HT network, high NBC values (> 0.100) were observed in only two taxa: *Trichocerca pusilla* and *C. cratera* (Table 3).

In the examined turbidity classes, similar trends were noted in EBC values denoting species that were bound by the strongest relationships to maintain the structure of the network. The main role was played by the above taxa and their interactions, including *C. vicinus*–*Polyarthra vulgaris* (MT; EBC = 143), *D. cucullata*–copepod nauplii (LT; EBC = 114), *Difflugia lobostoma*–*Synchaeta* sp. (HT; EBC = 59), and *Trichocerca pusilla*–*Anuraeopsis fissa* (HT; EBC = 50).

## Discussion

The application of graph theory to network analysis supported a detailed examination of interspecific interactions in the zooplankton network which were considerably influenced by the turbidity gradient. The model’s high taxonomic resolution enabled the identification of biomass flow as an indicator of interspecific relationships. Negative correlations involved mainly antagonist interactions between rotifers and, less frequently, predatory behavior of copepods and interference competition of cladocerans. In turn, positive correlations resulted mainly from the effect of feeding guilds and adaptation to environmental and feeding conditions in different turbidity classes.

A zooplankton network with a highly cohesive structure and strong interspecific interactions was observed in the HT class. Nodes with the highest closeness centrality (NCC > 0.6) were represented mainly by highly competitive species that actively search for food, i.e. raptorials *Polyarthra longiremis*, *Asplanchna priodonta* and *Cyclops vicinus* and filter-feeders *Daphnia cucullata* and *Bosmina longirostris*^34^. In the HT class, these taxa established strong correlations with detritivores, bacteriophages *Brachionus angularis*, *Hexarthra mira* and *Filinia longiseta*^51^, and psammophilous protozoa *Codonella cratera* and *Difflugia lobostoma*^52^. According to Boenigk and Novarino^25^, mineral suspension particles from mining operations promote the sedimentation of organic matter and the accumulation of biomass generated by producers, and they constitute an excellent substrate for the growth of algae, bacteria and protozoa. Similar observations were made in this study, where the content of chlorophyll *a* and suspended organic matter was higher, and protozoa were the most influential nodes (NCC) in the HT class than in the remaining turbidity classes. Therefore, the abundance of nutrients in HT class reservoirs could have been utilized by various trophic groups with different feeding strategies, without symptoms of competitive elimination^43^. Strong interspecific interactions with a high clustering coefficient were associated with an increase in biomass (positive correlations), which points to the coexistence of species that graze on phytoplankton (*P. longiremis* and *B. angularis*), detritus (*H. mira* and *Filinia longiseta*) and animal protein (*Asplanchna priodonta*)^51^. In turn, negative correlations between species were indicative of rotifer grazing on protozoa (*H. mira* vs. *Difflugia lobostoma*) and other rotifers (*A. priodonta* vs. *B. angularis*), copepod grazing on rotifers (*Thermocyclops crassus* vs. *K. valga*) or interspecific competition^50^.

Rotifers *P. longiremis* and *H. mira* established the highest number of interspecific relationships (NDC) characterized by the highest values of the correlation coefficient in the HT class. In the case of *P. longiremis,* the large number of neighbors could be attributed to its central location in the HT network that was most abundant in nutrients. Although *P. longiremis* is a common eurytopic species that tolerates a wide range of environmental conditions, it usually plays a dominant role in nutrient-rich environments^46,53–55^. In turn, sensory receptors in the mouth region of *H. mira* prevent the ingestion of inorganic particles, and they are an important feature that improves the species’ tolerance and adaptation to highly turbid environments^56^. Additionally, *Hexarthra mira* and, to a smaller extent, *P. longiremis* are anatomically adapted for rapid movement, which enables them to evade predation and interference competition from Cladocera^57,58^. Therefore, *H. mira* was an influential species mainly on account of its high motility, whereas evasive behavior enables the species to coexist with large cladocerans by minimizing the odds of being drawn into their branchial chambers^57^.

In a previous study by Goździejewska et al.^37^, the population size of the most influential “central” rotifer species, including *P. longiremis* and *H. mira*, was negatively (decrease in abundance) correlated with the physical and chemical parameters of suspended particles in HT class reservoirs. The current study also revealed that the average biomass (µg/l) of species with the highest NCC values was lower in the HT than the MT class, which suggests that high turbidity is a limiting abiotic factor. However, Goździejewska et al.^37^ relied on the results of multifactorial analyses to demonstrate that zooplankton (taxa and/or functional groups) respond differently to high turbidity, which is manifested in different changes in their species composition and functional feeding traits. In the present study, the network graph analysis revealed the presence of a cohesive network of interactions between species, which is indicative of directed biomass flow under high turbidity conditions. Kruk et al.^14^ also observed that network cohesion and the strength of interspecific relationships increased with a rise in the environmental salinity gradient.

High turbidity is associated not only with greater nutrient availability, but it also protects plankton against fish predation, which can contribute to high cladoceran biomass^36^. However, Cladocera played a less influential role (NCC) in the HT network than Copepoda (mainly *Cyclops vicinus* and copepodites) or Rotifera, which could be attributed to the fact that the analysed reservoirs are also used for recreational fishing and are regularly stocked with fish^46^. According to the size efficiency hypothesis^59^, the predatory behavior of planktivorous fish significantly affects size (large species and individuals are eliminated), species composition (Cladocera are eliminated due to their high energy value, lower motility and lower ability to evade predators)^35^ and interspecific interactions in zooplankton communities.

High turbidity also decreased the taxonomic diversity of zooplankton, and quantitative parameters (*H*′*, J*) were significantly lower in the HT than the MT and TL classes. These findings are explained by the intermediate disturbance hypothesis^60^ which states that local species diversity is minimized at high levels of disturbance because only adapted organisms survive, whereas less competitive species are eliminated. In the present study, the MT network was characterized by the highest zooplankton abundance and biomass and the highest number of species responsible for biomass flow, which confirms the intermediate disturbance hypothesis.

The most influential nodes in the MT network were represented by the same species that were responsible for the centrality of the HT network, but the closeness centrality measure (NCC), namely the strength of the correlations established by these nodes, decreased markedly (NCC < 0.5). The influence of path parameters between the most influential taxa (NBC) increased in the MT network which was composed of three distinct sub-networks (clusters). According to Kruk et al.^14^, the disintegration of a biocenotic network into clusters or sub-networks results from the absence of nodes (species) with high NCC values. The current study demonstrated that high network cohesion in the examined turbidity classes was determined by the threshold value of NCC (0.6), observed under high turbidity conditions. Interestingly, the number and strength of antagonistic interactions decreased (as demonstrated by the increase in the biomass of most species) in the entire MT biocenotic network relative to HT and LT networks, which points to independent feeding behavior^50^. Two sub-networks were characterized by strong positive correlations between raptorial feeders, mostly adult and juvenile stages of Cyclopoida. The third cluster featured mainly positive correlations between Rotifera species. Various taxonomic and trophic groups, i.e. *Bosmina longirostris* (Cladocera, large microphagous), *Cyclops vicinus* (Copepoda, raptorials) and *Polyarthra vulgaris* (Rotifera, raptorials), most of which were bound by positive relationships, played a key role in the communication between individual clusters (highest values of NBC and EBC), and determined the functioning of the MT network.

Martín González et al.^61^ emphasized the role of species with high NCC and NBC attributes because the network disintegrates more rapidly when these taxa are selectively removed from its structure. The above mechanism corresponds to the identification of keystone species that determine the species structure of biocenoses^62^. However, antagonistic predatory relationships are necessary to maintain the interspecific cohesion of systems, in particular those subjected to environmental changes^63^. In this study, the percentage of such relationships was low in the MT network.

Species with high NBC values also significantly influenced the LT network which was characterized by lowest density and highest heterogeneity (fragmentation). The LT network was composed of two overlapping clusters; therefore, the network centralization parameter was higher than in the MT class. *Daphnia cucullata* made the greatest contribution to the network’s cohesion (NCC = 0.512) and interspecies communication (NBC = 0.341). This species was positively correlated with non-competitive taxa, i.e. predatory rotifers (*Asplanchna priodonta*, *Synchaeta* spp.) and copepod nauplii (the most important relationship for maintaining the LT network; EBC = 114). *D. cucullata* was negatively correlated with microphagous rotifers (*Keratella tecta*, *K. valga*, *Brachionus angularis*) that are less effective filter feeders and are suppressed by large *Daphnia* through exploitative competition for shared food resources and through mechanical interference^64–66^. However, the LT environment featured the highest number of coexisting rotifer and cladoceran species, and it was characterized by the highest coefficients of taxonomic diversity in comparison with more turbid environments. In the above-cited studies, the size of *Daphnia* populations was directly correlated with their impact on the structure of Rotifera. However, it should be noted, that in the LT network, the average biomass of *D. cucullata* was more than 100 times lower and 250 times lower than in HT and MT networks, respectively. The above suggests that the influence exerted by *D. cucullata* on the LT environment was disproportionately high relative to its biomass. According to Ladle and Whittaker^67^, such species generally exert a strong influence on other organisms in the ecosystem and play an important role in the structure of zooplankton communities. The high values of both node centrality attributes indicate that *D. cucullata* is a species of demonstrable importance for ecosystem function^68^ and a keystone species in the LT network.

In summary, the application of network graph analysis enabled the identification of many phenomena and relationships in planktonic communities that have not been previously described in anthropogenic ecosystems. The applied methods elucidated the role played by taxa in the biocenotic network and the ecological mechanisms that are difficult to identify and interpret with the use of conventional structural and multidimensional analyses, in particular in in situ studies.

The influence of water turbidity on the interactions between zooplankton species has been rarely investigated in environmental research and appears to be undervalued. The present study demonstrated that the turbidity gradient considerably affects the structure of zooplankton communities. In high turbidity (HT) conditions, the species interaction network was characterized by the highest cohesion and the highest centrality attributes of taxa multidirectionally utilizing shared and abundant food resources. The structure of the network relied on equivalent significant positive and negative relationships that were controlled by five nodes (species) with very high values of centrality attributes (NCC > 0.6). Despite the fact that the physical and chemical attributes of turbid waters exert an inhibitory effect on zooplankton^37^, the biocenotic network created under high turbidity conditions was stable and highly functional. A decrease in water turbidity led a decrease in centralization attributes, and MT and LT networks were disintegrated into clusters (sub-networks). The significance of taxa influencing interspecies communication and biomass flow between sub-networks increased, but it also increased the risk that the loss of even one taxon could undermine the cohesion of the entire network.

In the view of the Remane^69^ hypothesis and the results by Kruk et al.^14^ about changes in zooplankton structure in the salinity gradient, as well as the observations made by Schmitz and Trussel^63^ on the key role of antagonistic relations in system cohesion, our results confirm that the biocenotic network functionality is more compromised in conditions of moderate turbidity than under boundary conditions.

## Methods

### Study area

**Figure 4:**
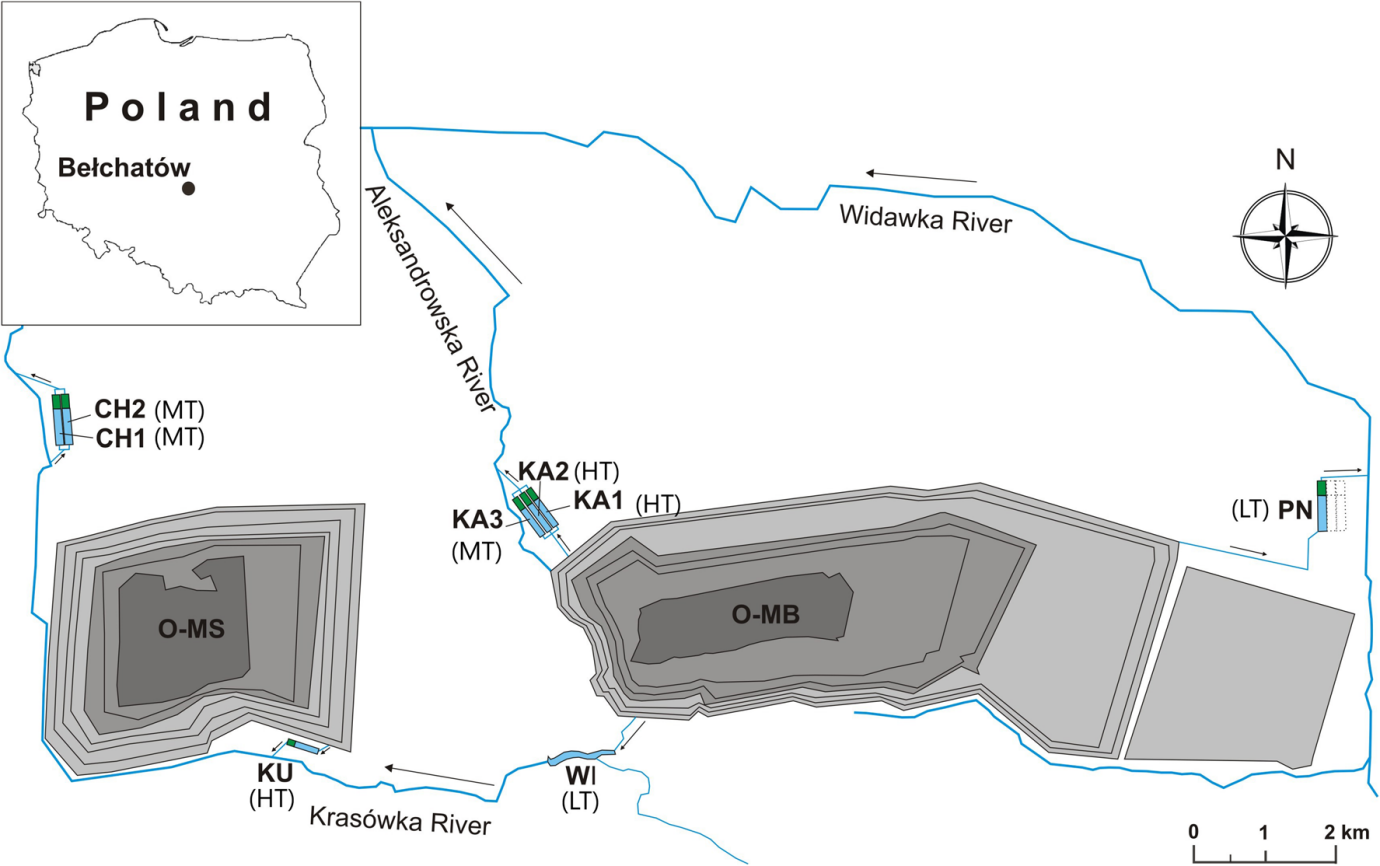
Location of the study area. *O-MB* opencast mine in Bełchatów, *O-MS* opencast mine in Szczerców; reservoirs CH1, CH2, and KA3 represent the MT class; reservoirs KA1, KA2, and KU represent the HT class; reservoirs PN, WI represent the LT class. Modified, see^37^.

The study was conducted in eight artificial reservoirs (CH1, CH2, KA1, KA2, KA3, KU, PN, WI) located in the vicinity of Bełchatów brown coal strip mine in Central Poland (51° 24′ 43.6″ N; 19° 26′ 32.9″ E). The studied reservoirs are part of the drainage network in Bełchatów and Szczerców coal strip mines (Fig. 4). Their main function is to reduce suspended matter through sedimentation^47,70^. The analysed mine pit lakes are flow-through reservoirs (with an estimated retention time of 16 h) with similar construction (three functional zones), shape, area (7.1–8.2 ha) and depth (1.7–2.7 m)^46,47^. However, water supplying the reservoirs originates from various depths and contains suspensions with different qualitative and quantitative traits, including mass concentration, as well as the size, shape and morphological structure of particles that determine turbidity levels^37^.

**Figure 5:**
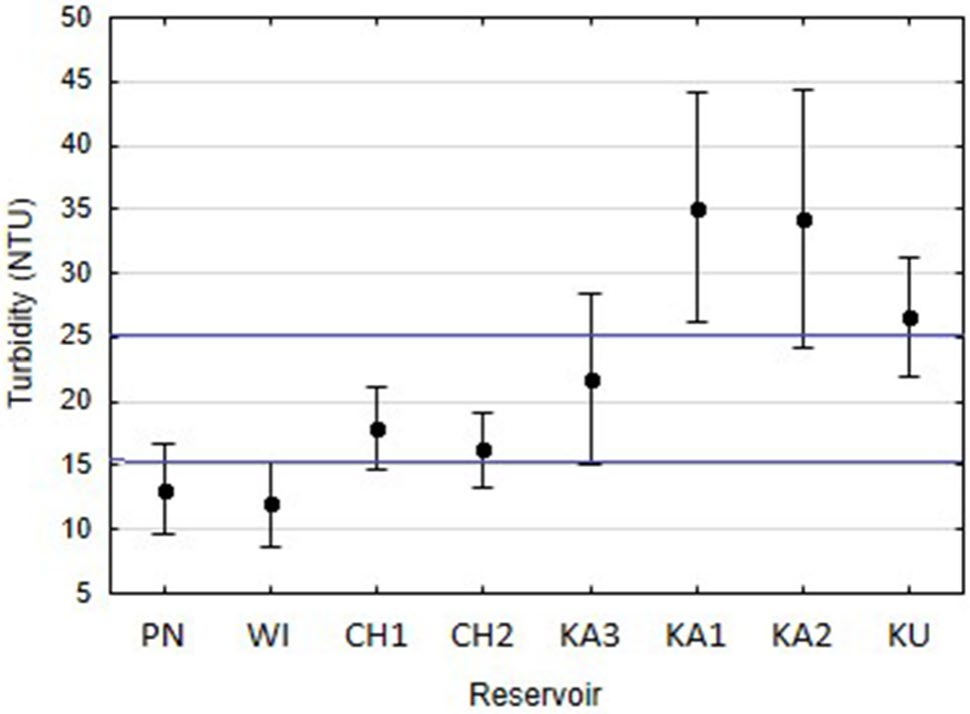
Water turbidity levels (mean ± SD) in 2014–2016, based on which the analysed reservoirs were divided into turbidity classes (HT, MT and LT).

To determine the influence of water turbidity on the species interaction network in zooplankton communities, the examined reservoirs were divided into three turbidity classes: high turbidity (HT) (> 25 NTU; KA1, KA2, KU), moderate turbidity (MT) (15–25 NTU; CH1, CH2, KA3), and low turbidity (LT) (< 15 NTU; PN, WI) (Fig. 5). The HT class involved 27 species and 158 observations, the MT class—28 species and 180 observations, and the LT class—22 species and 136 observations.

### Sampling and analytical procedure

Zooplankton were sampled monthly, between March and October of 2014 and 2015, and between June and September of 2016. Three sampling sites were located in the central part of each reservoir: directly in the center, in the coastal zone and in the vicinity of the filter zone. Samples were collected with a 5 l Patalas trap at a depth of approximately 1 m. During the experiment, a total of 480 zooplankton samples were collected, including 60 samples from each of the eight reservoirs. The collected samples with a volume of 20 l were filtered through a plankton net with 30 μm mesh size, preserved with Lugol’s solution, and fixed in 4% formalin solution. Zooplankton were identified to the lowest possible taxonomic level (with the exception of juvenile Copepoda stages) under a Zeiss AXIO Imager microscope, using the methods see^51,52,71–74^. In quantitative analyses, zooplankton abundance (ind/l) was determined with a Sedgewick-Rafter counting chamber. Zooplankton biomass (mg/l) was determined according to the methods see^75,76^. Species diversity (Shannon diversity index, *H*′), and species evenness (Pielou’s evenness index, *J*′) were analysed with the use of MVSP 3.22 software^77^.

The physical and chemical parameters of water were analysed in each zooplankton sampling site during each sampling event. Water temperature (°C), pH and dissolved oxygen (DO, mg/l) were measured with the YSI 6600 V2 Multi-Parameter Water Quality Sonde. Water transparency (SDT, m) was determined with the Secchi disk. Water samples were collected for laboratory analyses of color (Hazen scale), turbidity (NTU), total nitrogen (TN, mg/l), total phosphorous (TP, mg/l) and chlorophyll *a* (Chl *a,* µg l). Total suspended solids (Tot susp, mg/l) and the content of organic (Org susp, mg/l) and inorganic (In susp, mg/l) fractions were determined. Hydrochemical analyses were conducted in accordance with APHA guidelines^23^ (Table 1).

### Statistical and network analyses

The overall differences in the physical and chemical parameters of water and zooplankton parameters across the analysed turbidity classes were determined by one-way ANOVA (f, *P* ≤ 0.05) and post-hoc Tukey test (HSD). The non-parametric Kruskal–Wallis test (H, *P* ≤ 0.05) was used to determine the differences in zooplankton biomass between individual turbidity classes. (Statistica 13.0 for Windows, Statsoft, Tulsa).

In graph theory, the connections (edges) between objects (nodes) are examined by analyzing the parameters of the entire network and by determining the extent to which the attributes of individual nodes and edges affect the network and centrality measures^78^. In the present study, graph theory was applied to network analysis to compare the parameters of the zooplankton network in three turbidity classes and to determine the significance of individual species and interspecific interactions in these networks. The interactions between zooplankton species in three turbidity classes were analysed in the Cytoscape platform (http://www.cytoscape.org/) with the use of MetScape and NetworkAnalyzer applications to determine the correlations between data points. The database was composed of three csv files containing information about zooplankton data collected during period of the study in three turbidity classes. Zooplankton taxonomic units were listed in the columns, and the corresponding taxa biomass determined for every term of sampling in the rows. Data was normalized by autoscaling. The correlation matrix was computed in the Correlation Calculator 1.01 program (University of Michigan).

An undirected graph was created to identify all positive and negative interactions between zooplankton species in three turbidity classes. Positive interactions denoted co-occurrence patterns or mutualistic relationships between the biomass of zooplankton taxa, whereas negative interactions denoted predatory or competitive relationships^13^. The ranges of correlation coefficients for the edges were set so as to ensure that they were significant at *P* ≤ 0.05 for sample size in each turbidity class. The edge-weighted spring embedded layout was used with correlation coefficients as weights and weight-based heuristics. The absolute values of the correlation coefficients between nodes were used as weights. In weighted graphs, the distance between nodes is defined as the sum of weights^79^. In the distribution algorithm, network nodes are regarded as physical objects that repel each other, such as electrons. The connections between nodes (edges) are regarded as metal springs attached to a pair of nodes. The springs (edges) repel or attract nodes according to the power function (correlation). Nodes are positioned by the algorithm to minimize the sum of forces (correlations) in the network^79^.

The zooplankton network in three turbidity classes was compared based on the key network attributes that are applied in ecological studies, including the number of neighbors, closest path, clustering coefficient, network centralization, network density and network heterogeneity^13,80^. Three popular node centrality attributes and one edge attribute were used to determine the significance of zooplankton taxa in three turbidity classes: node degree centrality (NDC)^80^, node closeness centrality (NCC)^81^, node betweenness centrality (NBC) and edge betweenness centrality (EBC)^82^. Node closeness centrality measures the rate at which information is spread from a given taxon to other reachable taxa in the network, whereas NBC denotes the extent to which a given taxon contributes to the network’s cohesion. Edge betweenness centrality represents the significance of interspecific relationships for the integrity of the zooplankton network.

## Supplementary Information

Supplementary Tables.
