# Author Correction: Zooplankton network conditioned by turbidity gradient in small anthropogenic reservoirs

**DOI:** 10.1038/s41598-022-10387-6

**Published:** 2022-04-15

**Authors:** Anna Maria Goździejewska, Marek Kruk

**Affiliations:** 1 Faculty of Geoengineering, University of Warmia and Mazury, Oczapowskiego 5, 10-719 Olsztyn, Poland; 2 Faculty of Mathematics and Computer Science, University of Warmia and Mazury in Olsztyn, Słoneczna 54, 10-710 Olsztyn, Poland

Correction to: *Scientific Reports* 10.1038/s41598-022-08045-y, published online 10 March 2022

The original version of this Article contained an error in the Acknowledgments section.

“Project financially supported by Minister of Science and Higher Education the range of the program entitled “Regional Initiative of Excellence” for years 2019–2022, project No. 010/RID/2018/19, amount funding 12.000.000 PLN. This research was substantially funded by the PGE Górnictwo i Enegetyka Konwencjonalna SA Oddział KWB Bełchatów (agreement No. LPU/1225/2011), WFOSiGW in Łódź, and University of Warmia and Mazury in Olsztyn (18.610.010-110).”

now reads:

“Project financially supported by Minister of Education and Science the range of the program entitled “Regional Initiative of Excellence” for years 2019–2022, project No. 010/RID/2018/19, amount funding 12.000.000 PLN. This research was substantially funded by the PGE Górnictwo i Enegetyka Konwencjonalna SA Oddział KWB Bełchatów (agreement No. LPU/1225/2011), WFOSiGW in Łódź, and University of Warmia and Mazury in Olsztyn (18.610.010-110).”

The original Article has been corrected.

