# Supplementary material for: Zooplankton network conditioned by turbidity gradient in small anthropogenic reservoirs
Source: Sci Rep. 2022 Mar 10;12:3938. doi: 10.1038/s41598-022-08045-y (PMC8913641; doi:10.1038/s41598-022-08045-y)
Supplement: Supplementary file 1 — Supplementary Tables. [file 41598_2022_8045_MOESM1_ESM.docx]

Supplementary Material

**TITLE:**

**Zooplankton network conditioned by turbidity gradient in small anthropogenic reservoirs**

**Authors: Anna Maria Goździejewska & Marek Kruk**

Table S1. Species composition, biomass (µg L^-1^; mean ± SD) and zooplankton frequency (%) in individual turbidity classes. Values with the different superscripts are significantly different among reservoirs by non-parametric Kruskal–Wallis test (*P* ≤ 0.05).

| **Taxa** | **LT** | | | **MT** | | | **HT** | | | **Kruskal-Wallis** |
| --- | --- | --- | --- | --- | --- | --- | --- | --- | --- | --- |
|  | Biomass (µg L^-1^) | | Freq | Biomass (µg L^-1^) | | Freq | Biomass (µg L^-1^) | | Freq |  |
|  | 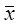 | ±SD | (%) | 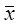 | ±SD | (%) | 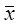 | ±SD | (%) | *P* |
| **Rotifera** |  |  |  |  |  |  |  |  |  |  |
| *Anuraeopsis fissa* | - | - | - | 0.003^a^ | 0.020 | 3 | 0.135^b^ | 0.783 | 13 | 0.0004 |
| *Ascomorpha ovalis* | 8.117^a^ | 22.965 | 38 | 14.55^b^ | 42.83 | 51 | 22.46^c^ | 48.95 | 66 | 0.0000 |
| *Ascomorpha saltans* | 0.001 | 0.009 | 1 | 0.648 | 6.655 | 2 | 0.074 | 0.513 | 3 | >0.05 |
| *Asplanchna priodonta* | 27.50 | 139.84 | 12 | 246.01 | 664.81 | 44 | 165.25 | 686.53 | 20 | 0.0000 |
| *Brachionus angularis* | 0.019^a^ | 0.094 | 4 | 1.408^b^ | 5.099 | 23 | 1.684^b^ | 14.484 | 17 | 0.0000 |
| *Brachionus calyciflorus* | 0.016^a^ | 0.189 | 1 | 3.649^b^ | 19.865 | 10 | 0.112^a^ | 0.781 | 3 | 0.0004 |
| *Brachionus diversicornis* | 0.005 | 0.058 | 1 | 0.019 | 0.183 | 1 | - | - | - | >0.05 |
| *Brachionus leydigii* | 0.731^a^ | 4.626 | 6 | 0.444^a^ | 3.812 | 2 | 0.038^b^ | 0.476 | 1 | 0.0213 |
| *Brachionus quadridentatus* | 0.073 | 0.489 | 2 | - | - | - | 0.032 | 0.397 | 1 | >0.05 |
| *Brachionus urceolaris* | - | - | - | 0.023 | 0.302 | 1 | 0.043 | 0.443 | 1 | >0.05 |
| *Cephalodella auriculata* | 0.020 | 0.145 | 2 |  |  |  | 0.002 | 0.030 | 1 | >0.05 |
| *Cephalodella gibboides* | 0.111^a^ | 0.553 | 9 | 0.054^b^ | 0.437 | 2 | 0.015^b^ | 0.118 | 2 | 0.0016 |
| *Cephalodella psammophila* | 0.019 | 0.082 | 7 | 0.005 | 0.043 | 1 | 0.010 | 0.080 | 2 | >0.05 |
| *Cephalodella* sp*.* | 0.074 | 0.166 | 28 | 0.020 | 0.086 | 7 | 0.076 | 0.514 | 12 | >0.05 |
| *Colurella colurus* | 0.054^a^ | 0.131 | 26 | 0.029^b^ | 0.147 | 9 | 0.022^b^ | 0.161 | 4 | 0.0000 |
| *Colurella uncinata* | 0.032 | 0.170 | 7 | 0.017 | 0.127 | 2 | - | - | - | >0.05 |
| *Conochilus unicornis* | 0.023 | 0.268 | 1 | 8.499 | 71.115 | 2 | - | - | - | >0.05 |
| *Dicranophorous sp.* | 0.061 | 0.508 | 1 | 0.068 | 0.653 | 2 | - | - | - | >0.05 |
| *Euchlanis contorta* | 0.461 | 3.416 | 5 | - | - | - | - | - | - | >0.05 |
| *Euchlanis dapidula* | 0.399 | 3.263 | 4 | 0.125 | 1.451 | 1 | 0.102 | 0.916 | 1 | >0.05 |
| *Euchlanis dilatata* | 0.636^a^ | 5.111 | 13 | 0.151^b^ | 0.944 | 3 | 0.100^b^ | 0.727 | 3 | 0.0001 |
| *Euchlanis lyra* | 0.302 | 2.508 | 3 | - | - | - | - | - | - | >0.05 |
| *Filinia longiseta* | 0.134 | 0.640 | 10 | 9.092 | 50.743 | 20 | 4.918 | 20.152 | 21 | >0.05 |
| *Filinia opolinensis* | - | - | - | - | - | - | 0.005 | 0.062 | 1 | >0.05 |
| *Hexarthra mira* | 0.054 | 0.318 | 4 | 4.861 | 18.527 | 16 | 1.299 | 7.725 | 8 | 0.0004 |
| *Kellicottia longispina* | - | - | - | 0.011 | 0.084 | 2 | 0.003 | 0.027 | 1 | >0.05 |
| *Keratella cochlearis* | 0.111^a^ | 0.260 | 33 | 9.554^b^ | 30.103 | 66 | 0.180^a^ | 0.393 | 34 | 0.0000 |
| *Keratella hiemalis* | - | - | - | 0.005 | 0.066 | 1 | - | - | - | >0.05 |
| *Keratella paludosa* | 0.004 | 0.051 | 1 | - | - | - | 0.003 | 0.032 | 1 | >0.05 |
| *Keratella quadrata* | 0.323^a^ | 1.249 | 19 | 7.070^b^ | 26.17 | 45 | 0.754^a^ | 4.203 | 22 | 0.0000 |
| *Keratella tecta* | 2.447^a^ | 12.101 | 28 | 16.48^b^ | 59.53 | 49 | 0.384^a^ | 1.343 | 31 | 0.0000 |
| *Keratella testudo* | 0.081 | 0.508 | 6 | 0.097 | 0.859 | 4 | 0.043 | 0.194 | 7 | >0.05 |
| *Keratella ticinensis* | - | - | - | 0.059 | 0.507 | 3 | 0.001 | 0.009 | 1 | >0.05 |
| *Keratella valga* | 0.081^a^ | 0.330 | 17 | 9.996^b^ | 39.156 | 39 | 3.726^b^ | 17.182 | 30 | 0.0000 |
| *Lecane arcula* | 0.0001 | 0.002 | 1 | 0.0001 | 0.006 | 1 | - | - | - | >0.05 |
| *Lecane bulla* | 0.037 | 0.129 | 10 | 0.032 | 0.236 | 3 | 0.032 | 0.161 | 6 | >0.05 |
| *Lecane closterocerca* | 0.004 | 0.023 | 4 | 0.001 | 0.008 | 1 | 0.001 | 0.012 | 1 | >0.05 |
| *Lecane cornuta* | 0.005 | 0.060 | 1 | - | - | - | - | - | - | >0.05 |
| *Lecane flexilis* | 0.001 | 0.006 | 1 | 0.003 | 0.023 | 2 | 0.003 | 0.019 | 2 | >0.05 |
| *Lecane furcata* | - | - | - | 0.0001 | 0.006 | 1 | - | - | - | >0.05 |
| *Lecane hamata* | - | - | - | 0.006 | 0.070 | 1 | 0.002 | 0.017 | 2 | >0.05 |
| *Lecane imbricata* | 0.000 | 0.005 | 1 | 0.001 | 0.008 | 1 | - | - | - | >0.05 |
| *Lecane inermis* | 0.001 | 0.012 | 1 | 0.002 | 0.015 | 1 | 0.0001 | 0.003 | 1 | >0.05 |
| *Lecane levistyla* | 0.002 | 0.023 | 1 | - | - | - | 0.002 | 0.022 | 1 | >0.05 |
| *Lecane ludwigii* | 0.001 | 0.011 | 1 | - | - | - | - | - | - | >0.05 |
| *Lecane luna* | 0.044^a^ | 0.170 | 8 | 0.016^b^ | 0.126 | 2 | 0.026^ab^ | 0.165 | 3 | 0.0152 |
| *Lecane lunaris* | 0.018 | 0.060 | 9 | 0.015 | 0.083 | 3 | 0.010 | 0.055 | 3 | >0.05 |
| *Lecane pyriformis* | - | - | - | - | - | - | 0.001 | 0.008 | 1 | >0.05 |
| *Lecane quadridentata* | 0.025 | 0.141 | 4 | - | - | - | - | - | - | >0.05 |
| *Lecane tryphema* | 0.002 | 0.015 | 1 | - | - | - | - | - | - | >0.05 |
| *Lepadella ovalis* | 0.028^a^ | 0.088 | 15 | 0.051^b^ | 0.375 | 6 | 0.029^ab^ | 0.139 | 9 | 0.0343 |
| *Lepadella patella* | 0.001 | 0.008 | 1 | 0.002 | 0.027 | 1 | 0.002 | 0.021 | 1 | >0.05 |
| *Lepadella rhomboides* | 0.008 | 0.047 | 3 | 0.018 | 0.170 | 1 | 0.089 | 0.665 | 5 | >0.05 |
| *Monommata maculata* | 0.113^a^ | 0.372 | 13 | 0.073^b^ | 0.455 | 3 | 0.026^b^ | 0.181 | 3 | 0.0002 |
| *Mytilina mucronata* | 0.016 | 0.111 | 2 | - | - | - | - | - | - | >0.05 |
| *Notholca acuminata* | 0.157 | 0.681 | 9 | 0.252 | 1.062 | 8 | 0.141 | 0.842 | 4 | >0.05 |
| *Notholca labis* | 0.004 | 0.032 | 1 | - | - | - | - | - | - | >0.05 |
| *Notholca squamula* | 0.284^a^ | 0.966 | 24 | 0.830^bc^ | 4.627 | 12 | 0.111^b^ | 0.479 | 10 | 0.0019 |
| *Notomata* sp*.* | 0.007 | 0.077 | 1 | - | - | - | - | - | - | >0.05 |
| *Polyarthra longiremis* | 24.82^a^ | 178.80 | 41 | 188.03^b^ | 306.05 | 90 | 55.51^c^ | 127.24 | 78 | 0.0000 |
| *Polyarthra major* | 0.007 | 0.085 | 1 | 0.134 | 1.518 | 1 | - | - | - | >0.05 |
| *Polyarthra vulgaris* | 0.002^a^ | 0.028 | 1 | 14.69^b^ | 32.36 | 42 | 2.127^a^ | 15.107 | 10 | 0.0000 |
| *Pompholyx complanata* | 0.019 | 0.148 | 4 | 0.030 | 0.166 | 5 | 0.014 | 0.090 | 3 | >0.05 |
| *Pompholyx sulcata* | - | - | - | 1.196^a^ | 4.877 | 15 | 0.134^b^ | 0.664 | 8 | 0.0378 |
| *Proales* sp. | 0.276 | 0.910 | 14 | 0.324 | 2.021 | 8 | 0.226 | 0.758 | 12 | >0.05 |
| *Resticula* sp*.* | - | - | - | - | - | - | 0.023 | 0.165 | 2 | >0.05 |
| *Scaridium longicaudum* | 0.016 | 0.073 | 5 | - | - | - | - | - | - | >0.05 |
| *Squatinella rostrum* | 0.009 | 0.073 | 1 | - | - | - | - | - | - | >0.05 |
| *Synchaeta* sp*.* | 18.06^a^ | 90.28 | 24 | 85.34^b^ | 199.48 | 59 | 41.19^c^ | 137.41 | 42 | 0.0000 |
| *Testudinella carlini* | - | - | - | 0.017 | 0.213 | 2 | - | - | - | >0.05 |
| *Testudinella patina* | 0.079 | 0.313 | 8 | 0.032 | 0.280 | 2 | 0.069 | 0.562 | 3 | >0.05 |
| *Testudinella truncata* | - | - | - | - | - | - | 0.001 | 0.009 | 1 | >0.05 |
| *Trichocerca elongata* | 0.002 | 0.018 | 1 | - | - | - | 0.003 | 0.033 | 1 | >0.05 |
| *Trichocerca iernis* | 0.070^a^ | 0.386 | 5 | 0.002^b^ | 0.028 | 1 | 0.017^ab^ | 0.127 | 3 | 0.0365 |
| *Trichocerca intermedia* | 0.002 | 0.010 | 4 | - | - | - | 0.003 | 0.018 | 4 | >0.05 |
| *Trichocerca musculus* | 0.005 | 0.027 | 4 | 0.005 | 0.054 | 1 | - | - | - | >0.05 |
| *Trichocerca myersi* | 0.004 | 0.025 | 2 | - | - | - | 0.009 | 0.086 | 1 | >0.05 |
| *Trichocerca porcellus* | 0.015 | 0.097 | 4 | - | - | - | - | - | - | >0.05 |
| *Trichocerca pusilla* | 0.002^a^ | 0.013 | 3 | 1.614^b^ | 10.25 | 14 | 1.588^b^ | 11.71 | 8 | 0.0021 |
| *Trichocerca scipio* | 0.002 | 0.020 | 1 | - | - | - | 0.0001 | 0.005 | 1 | >0.05 |
| *Trichocerca rattus* | 0.002 | 0.029 | 1 | - | - | - | - | - | - | >0.05 |
| *Trichocerca similis* | 0.003 | 0.024 | 1 | 0.009 | 0.115 | 1 | 0.024 | 0.231 | 2 | >0.05 |
| *Trichocerca stylata* | - | - | - | 0.063 | 0.697 | 1 | - | - | - | >0.05 |
| *Trichocerca taurocephala* | - | - | - | 0.005 | 0.055 | 1 | - | - | - | >0.05 |
| *Trichocerca tenuior* | 0.030 | 0.130 | 9 | - | - | - | 0.020 | 0.102 | 6 | >0.05 |
| *Trichocerca tigris* | 0.097 | 0.888 | 7 | - | - | - | 0.021 | 0.115 | 4 | >0.05 |
| *Trichocerca vernalis* | 0.002 | 0.020 | 1 | - | - | - | - | - | - | >0.05 |
| *Trichocerca weberi* | 0.000 | 0.005 | 1 | - | - | - | - | - | - | >0.05 |
| *Trichotria pocillum* | 0.013 | 0.070 | 4 | 0.023 | 0.159 | 2 | 0.020 | 0.132 | 3 | >0.05 |
| *Trichotria tetractis* | 0.021 | 0.097 | 5 | - | - | - | 0.009 | 0.082 | 1 | >0.05 |
| **Cladocera** |  |  |  |  |  |  |  |  |  |  |
| *Acroperus harpae* | 0.147 | 1.044 | 2 | - | - | - | - | - | - | >0.05 |
| *Alona affinis* | 0.588 | 3.803 | 4 | - | - | - | - | - | - | >0.05 |
| *Alona costata* | 0.074 | 0.857 | 1 | - | - | - | - | - | - | >0.05 |
| *Alona guttata* | 0.147 | 1.044 | 2 | - | - | - | 0.478 | 4.928 | 1 | >0.05 |
| *Alona quadrangularis* | 0.724^a^ | 3.809 | 6 | 0.084^b^ | 1.121 | 1 | 0.096^b^ | 1.197 | 1 | 0.0016 |
| *Alona rectangula* | 0.074 | 0.857 | 1 | - | - | - | 0.255 | 2.519 | 1 | >0.05 |
| *Alonella nana* | 0.037 | 0.429 | 1 | - | - | - | - | - | - | >0.05 |
| *Bosmina longirostris* | 18.923^a^ | 75.024 | 29 | 188.63^b^ | 393.70 | 47 | 140.18^a^ | 428.13 | 33 | 0.0000 |
| *Ceriodaphnia quadrangula* | 0.368 | 2.555 | 2 | - | - | - | 1.115 | 10.137 | 2 | >0.05 |
| *Chydorus sphaericus* | 0.434 | 2.144 | 6 | 0.729 | 5.359 | 3 | 0.089 | 0.819 | 1 | >0.05 |
| *Daphnia cucullata* | 25.07^a^ | 75.96 | 22 | 6485.7^b^ | 11618.8 | 83 | 2853.7^c^ | 6277.3 | 69 | 0.0000 |
| *Daphnia longispina* | - | - | - | - | - | - | 5.701 | 50.63 | 1 | >0.05 |
| *Eurycercus lamellatus* | 0.368 | 4.287 | 1 | 1.117 | 14.949 | 1 | - | - | - | >0.05 |
| *Graptoleberis testudinaria* | 0.551 | 3.833 | 2 | - | - | - | - | - | - | >0.05 |
| *Ilyocryptus agilis* | - | - | - | 0.223 | 2.990 | 1 | - | - | - | >0.05 |
| *Leptodora kindtii* | - | - | - | 21.23 | 128.50 | 4 | 16.56 | 69.89 | 6 | >0.05 |
| *Pleuroxus truncatus* | 0.110 | 1.286 | 1 | 0.335 | 4.485 | 1 | - | - | - | >0.05 |
| *Polyphemus pediculus* | - | - | - | 0.223 | 2.990 | 1 | - | - | - | >0.05 |
| *Scapholeberis mucronata* | 0.294 | 3.430 | 1 | - | - | - | 2.038 | 15.555 | 2 | >0.05 |
| **Copepoda** |  |  |  |  |  |  |  |  |  |  |
| *Acanthocyclops robustus* | - | - | - | 1.257 | 9.284 | 2 | 4.618 | 33.95 | 4 | >0.05 |
| *Cryptocyclops bicolor* | - | - | - | 6.899^a^ | 26.313 | 8 | 17.82^b^ | 58.66 | 16 | 0.0000 |
| *Cyclops scutifer* | - | - | - | 0.363 | 3.517 | 1 | 0 | 0 | 0 | >0.05 |
| *Cyclops strenuus* | - | - | - | 9.553 | 33.361 | 9 | 14.67 | 56.69 | 11 | >0.05 |
| *Cyclops vicinus* | - | - | - | 16.82 | 63.40 | 9 | 27.99 | 88.60 | 16 | >0.05 |
| *Diacyclops crassicaudis* | 1.103 | 6.489 | 4 | - | - | - | - | - | - | >0.05 |
| *Eucyclops macruroides* | 0.221 | 2.572 | 1 | 0.503 | 5.003 | 1 | - | - | - | >0.05 |
| *Eucyclops speratus* | 1.324 | 8.845 | 3 | 0.168 | 2.242 | 1 | - | - | - | >0.05 |
| *Eudiaptomus graciloides* | - | - | - | 33.296^a^ | 214.00 | 5 | 0.764^b^ | 6.750 | 1 | 0.0073 |
| Harpacticoida | 0.331 | 2.014 | 3 | 0.461 | 3.657 | 2 | 0.573 | 4.457 | 2 | >0.05 |
| copepodites | 4.632^a^ | 14.24 | 25 | 248.51^b^ | 450.59 | 87 | 165.83^b^ | 281.55 | 77 | 0.0000 |
| *Metacyclops minutus* | - | - | - | 0.559 | 4.067 | 2 | 0.892 | 8.577 | 1 | >0.05 |
| *Microcyclops varicans* | 0.588^a^ | 6.860 | 1 | 3.240^b^ | 19.763 | 5 | 14.14^c^ | 43.13 | 11 | 0.0000 |
| nauplii | 1.027^a^ | 1.180 | 69 | 65.52^b^ | 83.04 | 98 | 44.17^b^ | 50.93 | 96 | 0.0000 |
| *Thermocyclops crassus* | - | - | - | 40.22 | 136.86 | 17 | 20.73 | 75.36 | 15 | >0.05 |
| **Protozoa** |  |  |  |  |  |  |  |  |  |  |
| *Arcella discoides* | 0.052^a^ | 0.132 | 35 | 0.007^b^ | 0.037 | 4 | 0.005^b^ | 0.023 | 6 | 0.0000 |
| *Centropyxis aculeata* | 0.010 | 0.033 | 13 | 0.011 | 0.051 | 6 | 0.054 | 0.575 | 7 | >0.05 |
| *Codonella cratera* | 0.008^a^ | 0.031 | 13 | 12.39^b^ | 42.43 | 59 | 18.36^b^ | 58.68 | 59 | 0.0000 |
| *Difflugia acuminata* | 0.002 | 0.013 | 2 | - | - | - | 0.003 | 0.020 | 3 | >0.05 |
| *Difflugia lobostoma* | 0.043^a^ | 0.128 | 20 | 5.541^b^ | 13.86 | 35 | 0.981^a^ | 6.712 | 18 | 0.0000 |
| *Difflugia pyriformis* | 0.003 | 0.019 | 3 | - | - | - | - | - | - | >0.05 |

Table S2. The most important interspecies relationships of zooplankton in compared turbidity classes.

| **Turbidity class** | **Ralationships between taxa** | | |
| --- | --- | --- | --- |
| **LT** | **Negative** | *Ascomorpha ovalis - Filinia longiseta* | -0.18 |
|  |  | *Asplanchna priodonta - Keratella cochlearis* | -0.182 |
|  |  | *Euchlanis dilatata - Keratella cochlearis* | -0.201 |
|  |  | *Keratella valga* - *Synchaeta* sp. | -0.202 |
|  |  | *Keratella quadrata - Keratella tecta* | -0.209 |
|  |  | *Bosmina longirostris - Keratella quadrata* | -0.256 |
|  |  | *Ascomorpha ovalis - Asplanchna priodonta* | -0.264 |
|  |  | *Codonella cratera - Keratella valga* | -0.278 |
|  |  | *Asplanchna priodonta - Brachionus angularis* | -0.284 |
|  |  | *Daphnia cucullata - Keratella tecta* | -0.31 |
|  |  | *Difflugia lobostoma - Euchlanis dilatata* | -0.374 |
|  |  | *Daphnia cucullata - Polyarthra longiremis* | -0.475 |
|  |  | *Bosmina longirostris - Keratella tecta* | -0.605 |
|  | **Positive** | *Asplanchna priodonta - Keratella valga* | 0.867 |
|  |  | *Polyarthra longiremis - Synchaeta* sp. | 0.853 |
|  |  | *Bosmina longirostris - Ascomorpha ovalis* | 0.809 |
|  |  | *Euchlanis dilatata - Hexarthra mira* | 0.767 |
|  |  | *Ascomorpha ovalis - Keratella tecta* | 0.737 |
|  |  | *Daphnia cucullata - Synchaeta* sp. | 0.582 |
|  |  | *Filinia longiseta - Keratella tecta* | 0.431 |
|  |  | *Difflugia lobostoma - Hexarthra mira* | 0.421 |
|  |  | copepodites - nauplii | 0.418 |
|  |  | *Daphnia cucullata - Asplanchna priodonta* | 0.379 |
|  |  | *Ascomorpha ovalis - Keratella quadrata* | 0.371 |
|  |  | *Arcella discoides - Difflugia lobostoma* | 0.369 |
|  |  | *Asplanchna priodonta - Keratella tecta* | 0.33 |
|  |  | *Codonella cratera - Asplanchna priodonta* | 0.312 |
|  |  | *Colurella colurus - Polyarthra longiremis* | 0.302 |
| **MT** | **Negative** | *Brachionus angularis - Polyarthra vulgaris* | -0.154 |
|  |  | kopepodit *- Keratella cochlearis* | -0.157 |
|  |  | *Brachionus angularis - Difflugia lobostoma* | -0.16 |
|  |  | *Cyclops strenuus - Polyarthra vulgaris* | -0.163 |
|  |  | *Thermocyclops crassus - Asplanchna priodonta* | -0.164 |
|  |  | *Bosmina longirostris -* copepodites | -0.169 |
|  |  | *Asplanchna priodonta - Codonella cratera* | -0.181 |
|  |  | *Cryptocyclops bicolor - Microcyclops varicans* | -0.182 |
|  |  | *Keratella valga - Codonella cratera* | -0.182 |
|  |  | *Keratella tecta - Codonella cratera* | -0.199 |
|  |  | *Keratella cochlearis - Trichocerca pusilla* | -0.209 |
|  | **Positive** | *Ascomorpha_ovalis - Notholca_squamula* | 0.672 |
|  |  | *kopepodit - Thermocyclops_crassus* | 0.539 |
|  |  | *Trichocerca_pusilla - Arcella_discoides* | 0.521 |
|  |  | copepodites - nauplii | 0.435 |
|  |  | *Filinia longiseta - Keratella valga* | 0.426 |
|  |  | *Cyclops vicinus - Microcyclops varicans* | 0.418 |
|  |  | *Codonella cratera - Polyarthra vulgaris* | 0.407 |
|  |  | *Keratella tecta - Trichocerca pusilla* | 0.398 |
|  |  | *Colurella colurus - Keratella quadrata* | 0.388 |
|  |  | *Daphnia cucullata - Keratella tecta* | 0.369 |
|  |  | nauplii *- Hexarthra_mira* | 0.366 |
|  |  | *Polyarthra vulgaris - Trichocerca pusilla* | 0.337 |
|  |  | *Keratella valga - Polyarthra vulgaris* | 0.323 |
|  |  | *Bosmina longirostris -* nauplii | 0.318 |
|  |  | *Bosmina longirostris - Codonella cratera* | 0.305 |
| **HT** | **Negative** | *Thermocyclops crassus - Keratella tecta* | -0.223 |
|  |  | *Hexarthra mira - Keratella valga* | -0.226 |
|  |  | *Cyclops vicinus - Microcyclops varicans* | -0.227 |
|  |  | copepodites *- Keratella valga* | -0.232 |
|  |  | copepodites *- Hexarthra mira* | -0.24 |
|  |  | *Bosmina longirostris - Difflugia lobostoma* | -0.262 |
|  |  | *Asplanchna priodonta - Brachionus angularis* | -0.268 |
|  |  | *Brachionus angularis - Codonella cratera* | -0.271 |
|  |  | *Filinia longiseta - Keratella valga* | -0.34 |
|  |  | *Polyarthra longiremis - Codonella cratera* | -0.367 |
|  |  | *Bosmina longirostris - Hexarthra mira* | -0.396 |
|  |  | *Hexarthra mira - Difflugia lobostoma* | -0.404 |
|  |  | *Polyarthra longiremis - Brachionus angularis* | -0.45 |
|  | **Positive** | *Asplanchna priodonta - Hexarthra mira* | 0.587 |
|  |  | *Polyarthra longiremis - Hexarthra mira* | 0.533 |
|  |  | *Polyarthra longiremis - Filinia longiseta* | 0.514 |
|  |  | *Colurella colurus - Trichocerca pusilla* | 0.488 |
|  |  | *Bosmina longirostris - Brachionus angularis* | 0.482 |
|  |  | *Codonella cratera - Keratella valga* | 0.468 |
|  |  | *Cyclops strenuus - Polyarthra vulgaris* | 0.464 |
|  |  | *Microcyclops varicans - Thermocyclops crassus* | 0.452 |
|  |  | *Polyarthra longiremis - Keratella valga* | 0.441 |
|  |  | *Brachionus angularis - Keratella valga* | 0.431 |
|  |  | *Asplanchna priodonta - Difflugia lobostoma* | 0.402 |
|  |  | *Ascomorpha ovalis - Synchaeta* sp. | 0.331 |
|  |  | copepodites *- Thermocyclops crassus* | 0.326 |
|  |  | *Daphnia cucullata - Cryptocyclops bicolor* | 0.326 |
|  |  | *Codonella cratera - Hexarthra mira* | 0.315 |
